# Seasonal Changes in the Soil Microbial Community Structure in Urban Forests

**DOI:** 10.3390/biology13010031

**Published:** 2024-01-05

**Authors:** Xin Wan, Runyang Zhou, Sian Liu, Wei Xing, Yingdan Yuan

**Affiliations:** 1 Jiangsu Academy of Forestry, Nanjing 211153, China; lkywanxin@163.com; 2 Jiangsu Yangzhou Urban Forest Ecosystem National Observation and Research Station, Yangzhou 225006, China; 3 College of Horticulture and Landscape Architecture, Yangzhou University, Yangzhou 225009, Chinasianliu@yzu.edu.cn (S.L.)

**Keywords:** urban forest, soil microbial, tree species, community diversity, seasonal changes

## Abstract

**Simple Summary:**

In this study, high-throughput sequencing was used to analyze the microbial community composition of six stands. The findings of this study revealed the seasonal variations in the microbial community of the bulk soil in urban forests, as well as the microbial community structure associated with different tree species. These results offer valuable insights for the selection and conservation of tree species within urban forest stands.

**Abstract:**

Urban forests play a crucial role in the overall health and stability of urban ecosystems. Soil microorganisms are vital to the functioning of urban forest ecosystems as they facilitate material cycling and contribute to environmental stability. This study utilized high-throughput sequencing technology to examine the structural characteristics of bacterial and fungal communities in the bulk soil of six different forest stands: *Phyllostachys pubescens* (ZL), *Metasequoia glyptostroboides* (SSL), *Cornus officinalis* (SZY), mixed broad-leaved shrub forest (ZKG), mixed pine and cypress forest (SBL), and mixed broad-leaved tree forest (ZKQ). Soil samples were collected from each forest stand, including the corners, center, and edges of each plot, and a combined sample was created from the first five samples. The results revealed that among the bacterial communities, ZKG exhibited the highest alpha diversity in spring, while ZL demonstrated the highest alpha diversity in both summer and autumn. Proteobacteria was the most abundant bacterial phylum in all six forest stand soils. The dominant fungal phylum across the six forest stands was identified as Ascomycota. Notably, the microbial community diversity of SBL bulk soil exhibited significant seasonal changes. Although ZL exhibited lower bacterial community diversity in spring, its fungal community diversity was the highest. The bulk soil microbial diversity of ZL and SSL surpassed that of the other forest stands, suggesting their importance in maintaining the stability of the urban forest ecosystem in the Zhuyu Bay Scenic Area. Furthermore, the diversity of the bulk soil microbial communities was higher in all six stands during spring compared to summer and autumn. Overall, this study provides valuable insights into the seasonal variations of bulk soil microbial communities in urban forests and identifies dominant tree species, offering guidance for tree species’ selection and preservation in urban forest management.

## 1. Introduction

Soil microorganisms participate in several important ecological processes and are important components of forest ecosystems. Soil microorganisms can decompose litter [1,2], catalyze the turnover of soil carbon and nutrient elements [3], and are important drivers of biogeochemical processes [4]. Soil microorganisms are highly sensitive to environmental changes and are affected by various environmental factors. For example, soil microorganisms are very sensitive to changes in moisture conditions and temperature. An increase in moisture, continued warming, and earlier drought periods increase soil microbial biomass carbon and nitrogen [5]. Seasonal precipitation changes significantly increase the abundance of rare bacteria and dominant fungi in soil [6,7]. Studies have shown that seasonal changes affect the impact of nitrogen deposition on the soil microbial community structure, leading to varying responses of soil microorganisms to nitrogen deposition [8]. Soil microbial community characteristics show significant seasonal changes [9]. Soil temperature is the primary factor influencing seasonal variations in bacterial communities, and seasonal changes in soil temperature lead to changes in the composition of microbial communities. Bacteria, fungi, and actinomycetes are significantly positively correlated with soil temperature [10].

Urban forests are ecosystems composed of various woodlands as the main body, which can produce important ecosystem service effects, contribute to the improvement of environmental quality and sustainable urban development, and bring extensive benefits to urban residents. Urban forest construction can reduce the pressure and negative impact of urban development. Urban soil microorganisms are an important part of urban ecosystems and form the basis for maintaining their stability [11]. The soil microbial diversity is closely related to the environmental characteristics of soils [12]. Relevant studies have shown that soil pH is the most critical factor affecting changes in soil bacterial community [13]. Based on the exploration of soil microorganisms and soil physical and chemical properties in urban green spaces, it has been reported that soil microbial diversity is also affected by factors such as soil moisture and sand content [14]. With the intensification of human activities, urban soil pollution, particularly the impact of heavy metal ions on soil microorganisms, has become a common phenomenon. Research has shown that soil fungi are more susceptible to heavy-metal pollution than bacteria [15]. In addition, soil organic matter pollution can have an important impact on soil microbial diversity [16]. Urbanization is also an important reason for the loss of natural ecosystems and damage to the ecological environment [17]. Soil fungal diversity is expected to decrease as a result of urbanization, leading to the homogenization of soil fungal communities, whereas soil bacterial diversity is not significantly affected by urbanization [18].

As an important component of ecosystems, the correlation between soil microorganisms and environmental factors has always been an important topic in ecological research. Many researchers have extensively studied the correlations between environmental factors and soil microbial diversity. Environmental changes lead to changes in soil microbial diversity and species composition, and the soil microbial diversity is affected by various environmental factors. Related studies have found that, under drought conditions, soil fungal networks are more stable than soil bacterial networks [19]. Therefore, climatic conditions have a significant effect on the structure and diversity of the soil microbial communities. The community composition and function of the soil microbes are closely related to soil organic matter along latitudinal gradients [20]. These studies demonstrate that soil environmental conditions play an important role in microbial diversity and the community’s structural composition. Changes in land-use types in subtropical plantations affect the composition of soil microbial communities [21]. The addition of phosphorus to temperate grasslands exacerbates microbial nitrogen limitation and changes the composition and life-history strategies of microbial communities [22]. Therefore, the relationship between environmental factors and soil microorganism changes owing to changes in the living environment, and the effects of environmental factors on microorganisms are different in different ecological environments.

Urban forests play an important role in maintaining the stability of urban ecosystems. Six different forest stands were selected from the Zhuyu Bay Scenic Area in Yangzhou City, Jiangsu Province, China: SBL, SSL, SZY, ZKG, ZKQ, and ZL. Bulk soil samples were collected from various forest stands, and high-throughput sequencing analysis was conducted to study the diversity of soil microorganisms across different seasons. The purpose of this study was to investigate the seasonal changes of bulk soil microbial communities in urban forests and the composition of bulk soil microbial communities in different tree species. Here, we mainly focused on three questions: (1) what are the similarities and differences in the microbial community composition of the bulk soil of six different forest stands? (2) What is the relationship between the season and bulk soil’s microbial community composition? (3) Which tree species are more suitable as dominant tree species in urban forest ecosystems? Understanding these three questions will help further explore the significance of the interaction mechanism between plants, soil, and microorganisms in maintaining the stability of urban ecology.

## 2. Materials and Methods

### 2.1. Study Site and Experimental Design

Zhuyu Bay Scenic Area is located in the Guangling District of Yangzhou in the Jiangsu province. There is a National Positioning Observation and Research Station of Urban Ecosystem in Yangzhou City, Jiangsu Province. In this area, the climate transitions from subtropical to temperate monsoon, with an average altitude of two meters, average temperature of 15.8 °C, and average precipitation of 864 mm. In this study, six different stands were selected in Zhuyu Bay Scenic area: SBL, SSL, SZY, ZKG, ZKQ, and ZL. Soil samples from different stands were collected for high-throughput sequencing analysis to study the diversity of soil microorganisms in different seasons. The average temperature in spring, summer, and autumn is 15 °C, 28 °C, and 18 °C, respectively.

### 2.2. Sample Collection

Bulk soil (BS) refers to soil that is not near the roots of a selected plant. Each plot was sampled at five points: the corners, center, and edges. The sixth repeat was obtained by mixing the previous five samples. A soil auger was used to collect soil samples from depths of 0–20 cm. The samples were then examined to remove any litter, fine roots, small stones, or other impurities, and they were thoroughly mixed to reduce heterogeneity. Each sample was packaged in a sterile plastic bag, sealed, and then transported in ice to the laboratory. The samples were stored in a refrigerator at −80 °C for microbial sequencing.

### 2.3. DNA Extraction, Amplification, and Sequence Analysis

Microbial DNA from 108 samples from three seasons (six forest stands, with six replicates per plot) was extracted using the CTAB and SDS methods [23]. DNA concentration and purity were tested on a 1% agarose gel. Depending on the concentration, DNA was diluted to 1 ng/μL with sterile water. Primer sets 515F (5′-GTGCCAGCMGCCGCGG-3′) and 806R (5′-GGACTACHVGGGTWTCTAAT-3′) were used to generate bacterial libraries with a unique 6-nt barcode at the 5′-end of the forward primer to amplify the V4 region of the 16S rRNA gene for each sample. Similar to the process used to construct the bacterial library, the ITS1 region of the fungus was amplified using ITS1-1F-F (CTTGGTCATTTAGGAAGTAA) and ITS1-1F-R (GCTGCGTTCTTCATCGATGC) [24]. Sequencing libraries were generated, and index codes were added using the TruSeq^®^ DNA PCR-Free Sample Preparation Kit (Illumina, San Diego, CA, USA) according to the manufacturer’s instructions. The library quality was evaluated on a Qubit@ 2.0 fluorometer (Thermo Scientific, Waltham, MA, USA) and an Agilent Bioanalyzer 2100 system. After purification and quantification, the libraries were sequenced on an Illumina NovaSeq 6000 platform (Personalbio Company (Shanghai, China)) according to standard protocols. All raw data from the 16S V4 bacterial region and the fungal ITS1 region were processed using QIIME for quality control processes (V1.9.1, http://qiime.org/scripts/split_libraries_fastq.html, accessed on 1 October 2023) and FLASH for paired reads (V1.2.7, http://ccb.jhu.edu/software/FLASH/, accessed on 1 October 2023) [25,26]. For bacteria and fungi, annotation was performed by matching the Silva sequences with the UCHIME algorithm and Unite database (ITS: http://unite.ut.ee/, accessed on 1 October 2023) (UCHIME, https://www.drive5.com/usearch/manual/uchime_algo.html, accessed on 1 October 2023) [27,28]. 

### 2.4. Statistical Analysis

Alpha diversity was analyzed using three indices of species diversity: richness, Shannon’s evenness, and Pielou’s evenness. These indices were calculated and visualized using QIIME (Version 1.9.1) and the R package ggplot2. One-way ANOVA was used to determine significant differences at a *p*-value of 0.05, followed by Duncan’s post hoc test to identify values that were different. A beta diversity analysis was used to determine the differences in species complexity among samples, and Beta diversity on Bray–Curtis was calculated using QIIME software (version 1.9.1). To calculate the indicator values for each species in the comparison group, the R language Labdsv package (Version 2.1-0)was used [29].

## 3. Results

### 3.1. Alpha and Beta Diversities of Microbial Communities in Urban Forest Stands in Different Seasons

**Figure 1:**
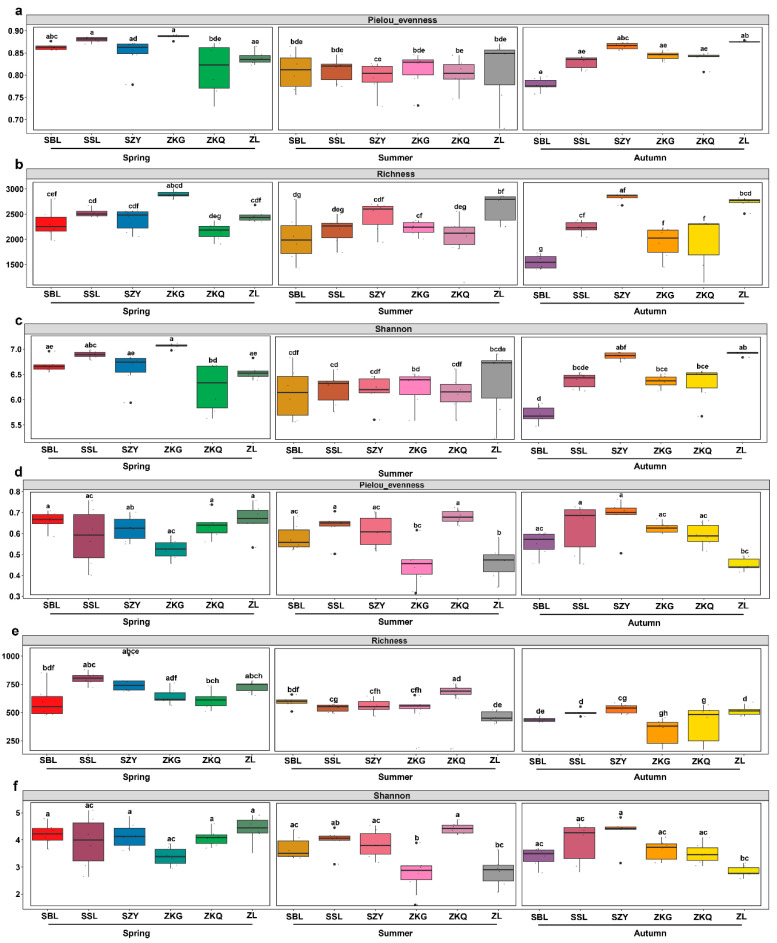
The variations in α diversity in the bulk soil of various city forest plots across three seasons. Pielou’s evenness (**a**,**d**), richness (**b**,**e**), Shannon index (**c**,**f**), bacterial (**a**–**c**) and fungal communities (**d**–**f**), The lower letters in the figure indicate the degree of difference between them.

To compare the diversity of microbial communities in the bulk soil of the urban forest, we conducted alpha diversity analysis for bacteria and fungi in different stands and seasons. Among the bacterial communities, the evenness of the Pielou index for ZKQ in spring was the lowest, indicating that the bacterial community in the bulk soil of ZKQ during spring had the lowest evenness. The evenness of the SBL in autumn was the lowest, and there were no significant differences among the other five forest stands. However, there was no significant difference in evenness between the six forest stands during the summer (Figure 1a). The results showed that forest stands with the highest richness of bulk soil bacterial communities also differed in different seasons. The highest richness was observed in ZKG in spring and ZL and SZY in summer and autumn, respectively (Figure 1b). In addition, for the Shannon index, the bulk soil bacterial community diversity of ZKG in spring, ZL in summer, and ZL and SZY in autumn was also the highest. Further research found that the evenness, richness, and diversity of urban-forest bulk soil bacterial communities were not significantly different between the six forest stands in spring and summer; however, the differences between the SBL and ZL in autumn were significant (Figure 1c). In the fungal community, the evenness of the Pielou index showed the same trend as that of the Shannon index (Figure 1d–f). The results showed that the evenness and diversity of the fungal community in the bulk soil of ZKG was relatively low in spring and summer. Similarly, the evenness and diversity of the fungal community in the bulk soil of ZL were relatively low in summer and autumn and several other forest types. The evenness and diversity of the fungal communities in the bulk soil were relatively high. Further research found no significant differences in evenness or diversity among the six forest stands in spring. The difference in evenness between ZL and ZKG was not significant in summer; however, the difference in evenness between ZL and the other four stands was significant. There was also a difference in the diversity between ZL and ZKQ. There were significant differences in evenness and diversity between ZL and SZY in autumn. Additionally, the difference in evenness between ZL and SSL was also statistically significant. For the richness index, the forest stands with the highest richness in each season were SSL in spring, ZKQ in summer, and SZY in autumn. There was a significant difference in richness between ZKG and ZKQ in spring and a significant difference in richness between ZL and SZY in summer and autumn. An alpha analysis of the bacteria and fungi revealed that the bacterial community had a higher evenness, richness, and diversity than the fungal community.

**Figure 2:**
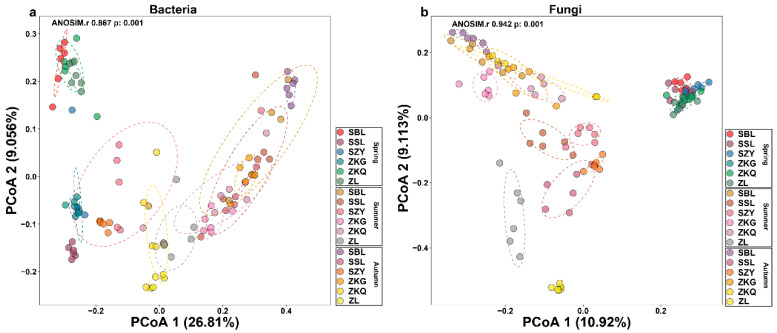
Analysis of β diversity of bulk soil microbial communities in different stands in three seasons. (**a**) Principal coordinate analysis (PCoA) of bacterial communities; (**b**) PCoA of fungal communities.

We also performed a beta diversity analysis on bacteria and fungi to compare the differences between the samples. The contribution values of PCoA1 and PCoA2 to the differences in bacterial communities in six forest soil samples across three seasons were 26.81% and 9.056%, respectively. Additionally, the contribution values of PCoA1 and PCoA2 to the differences in fungal communities in six forest soil samples across three seasons were 10.92% and 9.113%, respectively. Among the bacterial communities, the reproducibility of the six forest stands in spring was relatively good. SBL, ZKQ, and ZL were far from the other three forest stands, indicating that the bacterial community compositions in SBL, ZKQ, and ZL were relatively similar. The bacterial community compositions of the other three stands were significantly different. The distance between ZL and the other five forest stands in summer and autumn reflected the differences in bacterial communities between ZL and the other five forest stands (Figure 2a). In the fungal community, there were no significant differences in the bacterial communities among the six forest stands in spring, but there were large differences in the composition of the fungal community among the six forest stands in summer and autumn. Among them, ZL differed from the other five forest species in summer and autumn. Differences in bacterial communities were most significant among the forest stands. Further research found significant differences in the composition of bacterial communities in the same forest during different seasons, including bacterial and fungal communities (Figure 2b).

### 3.2. Microbial Community Composition in Urban Forest Stands in Different Seasons

**Figure 3:**
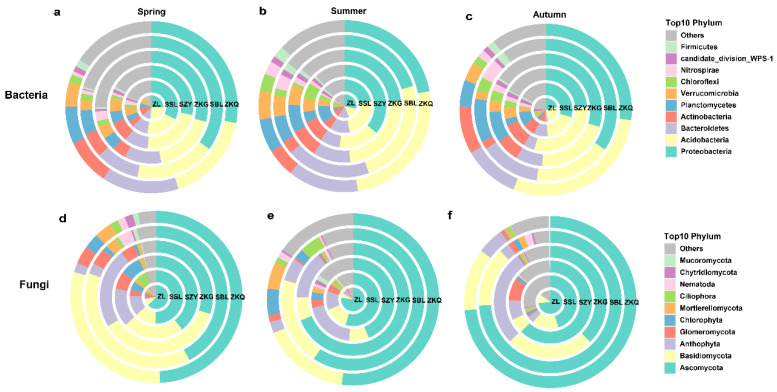
The relative abundance of the top 10 bacterial and fungal phyla in bulk soil in urban forest stands in the three seasons (see also Supplementary file). Relative abundance of bacterial (**a**–**c**) and fungal communities (**d**–**f**) in spring (**a**,**d**), summer (**b**,**e**), and autumn (**c**,**f**).

Among the OTU annotation results of bulk soil bacterial and fungal communities at the phylum level in six forest stands, the bacterial communities in spring, summer, and autumn were 32, 34, and 34 phyla, and the fungal communities were 24, 23, and 21 phyla, respectively. In the bacterial community, the dominant bacterial phyla with relatively high relative abundances in the six forest stands were Proteobacteria and Acidobacteria during the three seasons. The relative abundance of the dominant bacterial phyla in different seasons differed among the six forest stands. The relative abundance of Proteobacteria was highest in the SBL in spring and autumn, whereas the relative abundance of Proteobacteria in SZY was highest in summer. The relative abundance of Acidobacteria was highest in ZL during spring, SSL and ZKQ in summer, and ZKQ in autumn (Figure 3a–c). Among the fungal communities, the fungal community with the highest relative abundance was Ascomycota. In the three seasons, the relative abundance of Ascomycota was the highest in ZL. In autumn, the relative abundance of Ascomycota was relatively high in the SBL and ZKQ forest stands. The relative abundance of Ascomycota in ZKG was the lowest in both spring and autumn, whereas it was the highest in summer, except for ZL. In addition, the relative abundance of Basidiomycota was second only to that of Ascomycota. The relative abundance of Basidiomycota was higher in spring and lower in winter. However, the relative abundance of Basidiomycota in ZKG was higher in winter than in summer (Figure 3d–f). The results also show that the relative abundance of the same dominant bacterial phylum in different forest stands and that of different dominant bacterial phyla in the same forest stand were different. 

### 3.3. Relative Abundance of OTUs in Soil Microbial Communities during Different Seasons

**Figure 4:**
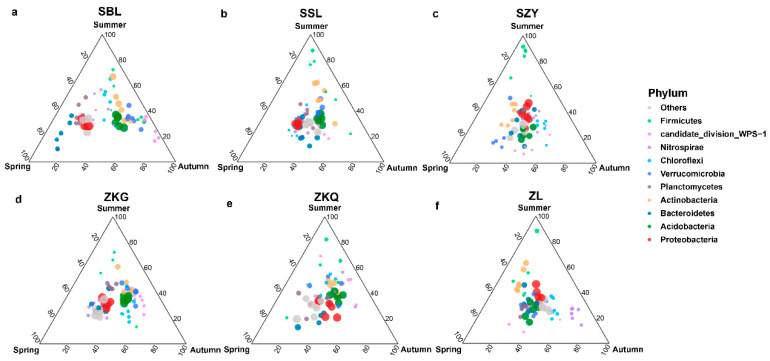
Ternary map of bulk soil bacterial community OTUs during different seasons. The size of each point indicates the relative abundance of OTUs. (**a**) SBL, (**b**) SSL, (**c**) SZY, (**d**) ZKG, (**e**) ZKQ, and (**f**) ZL.

At the phylum level, the dominant bacterial phyla in the six forest stands in the three seasons were Proteobacteria and Acidobacteria. The results showed that the bacterial community structures in the bulk soil of the six forest stands were different. Additionally, Proteobacteria in the SBL, SSL, and ZKG forest stands exhibited significant differences. Similar to the relative abundance of Acidobacteria in the three seasons, Proteobacteria had the highest relative abundance in spring, and Acidobacteria had the highest relative abundance in autumn. The relative abundance of Proteobacteria and Acidobacteria in SZY and ZL was similar across the three seasons. Proteobacteria had the highest relative abundance in summer, whereas Acidobacteria had the lowest relative abundance in summer. In addition, the relative abundance of both Proteobacteria and Acidobacteria in ZKQ was highest in autumn. In addition, the relative abundance of the bacterial community in the SBL changed with season. Proteobacteria and Bacteroidetes had the highest and lowest relative abundances, respectively, in spring and autumn. The relative abundance of Actinobacteria was higher in summer and autumn and lowest in spring. Acidobacteria and Verrucomicrobia had the highest relative abundances in autumn, followed by summer (Figure 4a–f).

**Figure 5:**
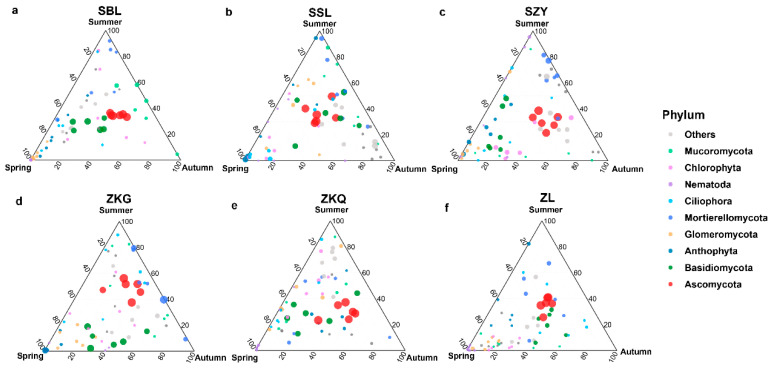
Ternary map of bulk soil fungal community OTUs in different seasons. The size of each point indicates the relative abundance of OTUs. (**a**) SBL, (**b**) SSL, (**c**) SZY, (**d**) ZKG, (**e**) ZKQ, and (**f**) ZL.

Among the fungal communities, the bulk soil community structure differed between seasons and forest stands. The dominant fungal phylum in the six forest stands was the Ascomycota. During the three seasons, the relative abundance of Ascomycota in SSL and ZL was not different, whereas the relative abundance of Ascomycota in the other four forest stands was different. The relative abundance of Ascomycota in SBL, SZY, and ZKQ was highest in autumn, and the relative abundance of Ascomycota in ZKG was highest in summer. In addition, the relative abundance of Basidiomycota in SBL, ZKG, and ZKQ first decreased and then increased from spring to summer to autumn, whereas the relative abundance of Basidiomycota in SZY gradually decreased from spring to summer to autumn (Figure 5a–f).

### 3.4. Taxonomic Tree and Bitaxonomic Network Analysis of Dominant Phyla of Soil Microbial Communities in Different Stands

**Figure 6:**
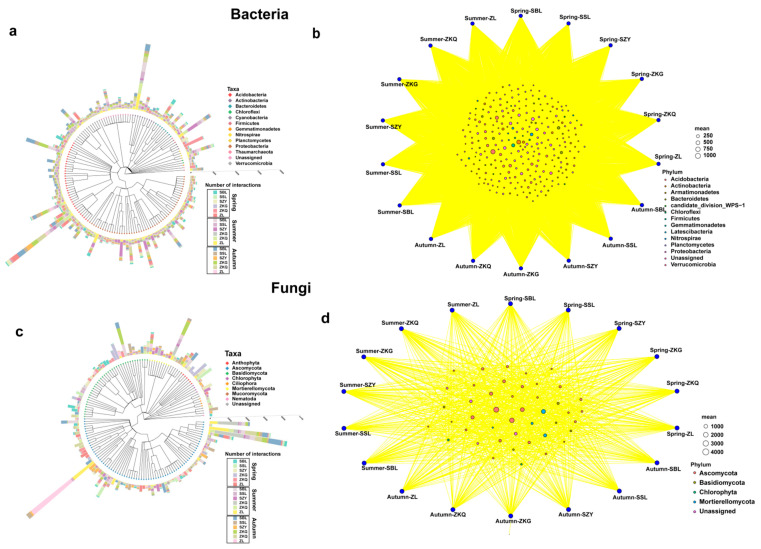
Species classification tree analysis (**a**,**c**) and dichotomous network analysis (**b**,**d**).

The results of the species classification tree and bipartite network analyses showed that the species richness of different bacterial phyla was also different. Proteobacteria was the phylum with the highest species richness, followed by Acidobacteria. Among them, the species evenness of ZKQ in spring, SZY in summer, and SBL in autumn was relatively low. The common bacterial phyla in the six forest stands in the three seasons mainly included Proteobacteria, Acidobacteria, and Bacteroidetes (Figure 6a,b). In the fungal community, Ascomycota had the highest species richness, followed by Basidiomycota. The uniformity of the ZL was relatively low in summer and autumn, and the uniformity of the ZKG was also relatively low in spring and summer. The fungal phyla shared by the six forest species in the three seasons were Ascomycota, Basidiomycota, Chlorophyta, and Mortierellomycota (Figure 6c,d).

## 4. Discussion

Bacteria and fungi are the main components of soil microorganisms and play important roles in nutrient cycling [30,31]. This study revealed the specific community structure of bulk soil bacterial and fungal communities in different forest stands. We found that different forest stands had an impact on bulk soil microbial diversity, which is consistent with previous research [32,33]. The OTU annotation results showed that the dominant bacterial phyla in the six forest stands were Proteobacteria, Acidobacteria, Bacteroidetes, Actinobacteria, Planctomycetes, Verrucomicrobia, Chloroflexi, Nitrospirae, and Firmicutes. The relative abundance in the bulk soil of the six forest stands was the highest. The main bacterial phyla were Proteobacteria and Acidobacteria. Many studies have shown that Proteobacteria are the main functional bacteria that decompose and transform litter [34,35]. Acidic soils are conducive to the growth and reproduction of acidophilic bacteria [36]. The dominant bacterial phyla in the fungal community were Ascomycota and Basidiomycota. The dominant fungal phylum with the highest relative abundance in the six forest stands was Ascomycota, which is consistent with the results of previous studies [37,38]. Many Ascomycota fungi grow and reproduce by absorbing the humus. The relative abundance of Basidiomycota in BL and ZKQ was high. Basidiomycota can decompose lignocellulose and is the main decomposer of soil fungi [39].

Bulk soil microbial community diversity is affected by season, and the relative abundance of microbial communities differs between seasons [40]. According to the alpha diversity analysis, we found that the diversity of bulk soil microbial communities in SBL changed significantly with season, with the highest diversity in spring, followed by summer, and the lowest diversity in autumn. Pine forests are at their peak metabolism in spring, which provides favorable conditions for the growth and reproduction of bacteria and fungi, resulting in higher microbial diversity. However, in autumn, the root systems of pine and forest trees enter a dormant period with less metabolic activity and lower microbial diversity. In addition, among the six forest stands, the difference in the microbial diversity of the bulk soil in spring was the least significant, but the diversity was higher. This may be because spring is the reproductive period for microorganisms, and the number and types of microorganisms in the soil increase.

Selecting tree species with a high bulk soil microbial community diversity is conducive to maintaining the stability of urban forest ecosystems. The results of this study showed that vegetation type has an impact on urban forest bulk soil microbial communities [32]. There have also been studies using MiSeq sequence analysis to assess the structure of microbial communities, revealing specific genetic structures of bacterial and fungal communities in similar soils under different tree species, and observing that tree species have a significant effect on soil microbial diversity [41]. Among the six forest stands, the bacterial Shannon index of ZL was highest in both summer and autumn, and the Shannon index in spring was relatively high, indicating that ZL had higher bacterial diversity. Relevant studies have shown that bamboo forests have more litter and nutrients for microorganisms [42]. This may be one of the reasons why the number of microorganisms in the bulk soil of bamboo forests is relatively high. Soil microorganisms promote plant growth by mineralizing soil organic matter and supplying available nutrients [43]. Further research found that ZKG and SSL in spring, ZL and ZKG in summer, and ZL and SZY in autumn showed a higher bacterial diversity. In the fungal community, ZL and SBL in spring, ZKQ and SSL in summer, and SZY and SSL in autumn showed a higher bacterial diversity. The diversity was high, among which ZL and SSL appeared with the highest frequency, indicating that the bulk soil microbial community structures in the ZL and SSL were relatively complex [41]. It can be used as a dominant tree species for maintaining the stability of urban forest ecosystems.

This study investigated the community structure and diversity of bulk soil bacteria and fungi in six forest stands in the Yangzhou Zhuyu Bay Scenic Area over three seasons. It not only revealed the impact of different seasons on the diversity of bacterial and fungal communities but also revealed the different effects of forest stands on microbial diversity. These results will help conduct in-depth research on urban forest soil microbial diversity in the future and are of great significance for maintaining the stability of the urban ecological environment.

## 5. Conclusions

This study revealed the effects of different tree species on bacterial and fungal community diversity. Among the six forest stands, ZL and SSL had the highest microbial community diversity and could be used as the dominant tree species in urban forests. In addition, this study revealed the response of microbial community structure to seasonal changes. We found that the diversity of bulk soil microbial communities in the SBL changed significantly with seasonal change, with the highest diversity in spring and the lowest diversity in autumn. Compared with summer and autumn, the bulk soil microbial community diversity in spring was higher among the six forest stands. Overall, this study revealed the seasonal changes of microbial community in bulk soil of urban forest and the microbial community structure of different tree species, providing guidance for the selection and protection of tree species in urban forest.

## Data Availability

Raw Illumina sequence data were stored for bacterial 16S and ITS fungal data in the archives of the NCBI Sequence Read with accession numbers PRJNA1029967 (Bacterial) and PRJNA1030501 (Fungal).

## Supplementary Materials

The following supporting information can be downloaded at: https://www.mdpi.com/article/10.3390/biology13010031/s1, Table S1: Top 10 Phylum.
